# Abstract not submitted for online publication

**DOI:** 10.1186/gb-2011-12-s1-i2

**Published:** 2011-09-19

## 

Abstract not submitted for online publication

